# Background noise analysis in urban airport surroundings of Brazilian cities, Congonhas Airport, São Paulo

**DOI:** 10.1590/S1518-8787.2016050006431

**Published:** 2016-11-24

**Authors:** Fabio Scatolini, Cláudio Jorge Pinto Alves

**Affiliations:** I Programa de Pós-Graduação em Engenharia de Infraestrutura Aeronáutica. Departamento de Engenharia Civil. Instituto Tecnológico da Aeronáutica. São José dos Campos, SP, Brasil

**Keywords:** Airports, Noise, Transportation, adverse effects, Noise Measurement, Sound Contamination, Urban Area

## Abstract

**OBJECTIVE:**

To perform a quantitative analysis of the background noise at Congonhas Airport surroundings based on large sampling and measurements with no interruption.

**METHODS:**

Measuring sites were chosen from 62 and 72 DNL (day-night-level) noise contours, in urban sites compatible with residential use. Fifteen sites were monitored for at least 168 hours without interruption or seven consecutive days. Data compilation was based on cross-reference between noise measurements and air traffic control records, and results were validated by airport meteorological reports. Preliminary diagnoses were established using the standard NBR-13368. Background noise values were calculated based on the Sound Exposure Level (SEL). Statistic parameters were calculated in one-hour intervals.

**RESULTS:**

Only four of the fifteen sites assessed presented aircraft operations as a clear cause for the noise annoyance. Even so, it is possible to detect background noise levels above regulation limits during periods of low airport activity or when it closes at night.

**CONCLUSIONS:**

All the sites monitored showed background noise levels above regulation limits between 7:00 and 21:00. In the intervals between 6:00-6:59 and 21:00-22:59 the noise data, when analyzed with the current airport operational characteristics, still allow the development of additional mitigating measures.

## RESUMO

**OBJETIVO:**

Avaliar quantitativamente o ruído de fundo no entorno do aeroporto de Congonhas, com base em ampla amostragem e medições sem interrupção.

**MÉTODOS:**

Locais de medição escolhidos a partir de curvas de ruído de 62 e 72 LDN (day-night level), em equipamentos urbanos de uso compatível com o residencial. Quinze locais foram avaliados por mais de 168 horas consecutivas cada um (sete dias). A compilação baseou-se em cruzamentos de dados do controle de tráfego aéreo e os resultados foram validados por meio de relatórios meteorológicos do aeroporto. Diagnósticos preliminares foram estabelecidos utilizando a NBR-13368. O ruído de fundo foi calculado com base no Sound Exposure Level (SEL). Os parâmetros estatísticos foram calculados em intervalos de uma hora.

**RESULTADOS:**

Apenas quatro dos 15 locais avaliados apresentaram clara contribuição da operação de aeronaves no incômodo. Mesmo assim, é possível identificar ruído de fundo acima do regulamentar durante os períodos de baixa atividade ou de fechamento do aeroporto durante a noite.

**CONCLUSÕES:**

Todos os locais avaliados apresentaram ruído de fundo acima do regulamentar entre 7:00h e 21:00h. Nos intervalos entre 6:00h-6:59h e 21:00h-22:59h, os dados de monitoramento, quando analisados em conjunto com as atuais características operacionais do aeroporto, ainda possibilitam a elaboração de medidas mitigadoras adicionais.

## INTRODUCTION

The noise generated by aircraft operating in urban airports causes great dissatisfaction and annoyance for people who live nearby. The indirect physiological effects of this anthropic activity, mainly those interfering in the nocturnal sleep, force important airports in Europe to interrupt their operations for a few hours during the night, such as the Frankfurt in Germany, the Gatwick in United Kingdom, among others. Competition between economic and environmental impacts is not rare^9^ ^,^ ^21^, leading airport authorities to try to concialiate both issues^12^ ^,^ ^13^ ^,^ ^16^.

Continuous exposure to average noise levels with intensity above 65 dB(A) can cause several psychophysiological disorders^10^, regardless of age, such as sleep disorders, decreased work performance, high blood pressure, and aggravation of vascular diseases^3^ ^,^ ^19^.

However, since noise emission limits became more restrictive for civil aircraft in 2001 by the International Civil Aviation Organization (ICAO)^a^, the non-aircraft noise, also known as “background noise”, has gained importance to determine diagnosis of noise pollution in airport surroundings, regarding the designing (or development) of the respective mitigating measures. In tropical countries such as Brazil, the intensity of urban noise in general can be very high^14^. In European countries, the opposite can be easily found^1^.

Although the aircraft noise may not, in the short term, be significantly reduced at the source, noise monitoring systems in airports are still a useful tool to improve environmental policies in airport administrations^20^. Such systems help the identification of daily critical periods related to the residents’ annoyance^17^, seeking to adjust curves, noise contours, or noise zoning plans, obtained by mathematical models and annoyance metrics^11^. They also help the air traffic control to develop trajectories of little annoyance^15^.

The metrics that determine sound annoyance caused by aircraft generally do not consider the background noise, which frequently results in the determination of the most problematic areas that are inconsistent with the local reality. In fact, even the document ICAO 9829^b^ that presents and describes the “Balanced Approach” concept – widely accepted and supported by all the actors involved in the problem – does not mention background noise. Thus, these discrepancies have been better managed with noise monitoring systems, whose associated compiled data and opinion surveys, applied to residents^8^, often provide a good view of the problem.

The objective of this study was to evaluate, in a more objective manner, the background noise related to urban airports operations in Brazil. We proposed a procedure to identify aircraft noise events, considering the total environmental noise and dismissing the use of surveys. The Congonhas Airport (São Paulo, SP) was used as a case study because, even being one of busier Brazilian airports, it remains closed for seven hours a day since 1995, working in the other hours of the day near to its maximum capacity. An alternative methodology was adopted (mobile collectors) for the necessary measurements in order to optimize both the equipment use and the sampling size, without affecting accuracy.

## METHODS

**Figure 1:**
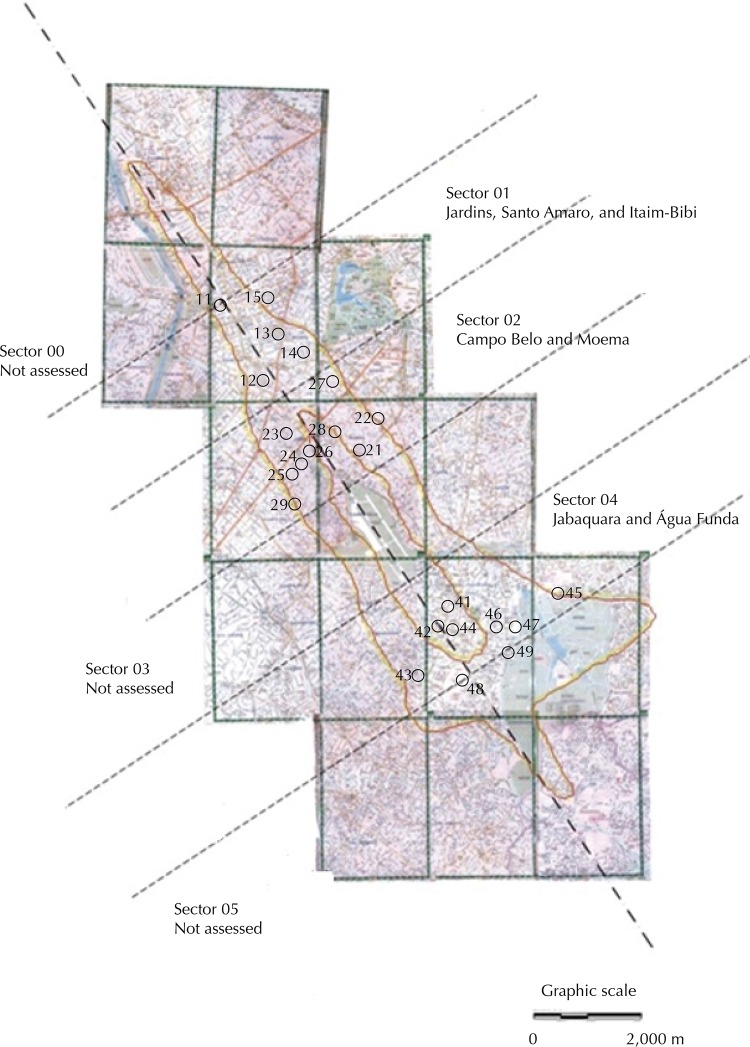
Noise contours for Congonhas Airport (62 and 72 DNL), and location of the 23 pre-selected sites for noise measurement.

Initially, the study’s range was established, from the Congonhas Airport, for monitoring purposes. Noise contours were used according to DNL metric (day-night-level), formally adopted by the National Civil Aviation Agency (ANAC) according to the Brazilian Civil Aviation Regulation 161 (RBAC 161). Using the Integrated Noise Model software (INM) of the Federal Aviation Administration, United States of America (FAA), two closed curves were calculated and drawn around the airport limits, representing the union of points with the same DNL (Figure 1).

The values of the internal and external curves are, respectively, 72 and 62 DNL. By the metric’s definition, these values are equivalent to simulated noise levels of 67.8 and 57.8 LEq (equivalent continuous sound level in dB(A)), respectively, in a period of 24 hours and produced exclusively by aircraft. These values (72 and 62 DNL), although different than what ANAC^c^ adopts for Noise Zoning Plans at airports, are more compatible with the national regulation^d^ ^,^ ^e^, which establish “significant” sound impact to measured values above 3 dB(A) from the allowed limit or the background noise (whichever is louder).

Figure 1 shows a map with noise contours produced for the study.

After the aviation accident occurred in July 2007 (TAM 3054), the allowed number of aircraft operations at the Congonhas Airport was limited to 34 per hour and can reach 40 in peak hours (early morning and late afternoon), although the infrastructure installed comprise up to 50 operations per hour. The INM was fed with relevant information about air traffic, with the intent to emulate the maximum capacity established by the regulations (210,000 operations/year), considering that 12.0% of the operations occur at night (between 22:00 and 6:59), because the airport closes every day at 23:00 and reopen at 6:00.

Twenty-three sites were pre-selected using maps that presented the detailed street layout and were visited before the beginning of measurements. Then, fifteen were chosen to receive the measuring equipment, taking particularly into account the prevalence of residential use on the surroundings and the potential exposure to noise. The perimeter of 62 DNL was subdivided into six “sectors”, drawing five lines orthogonal to the airport runway axis, distant each other by one runway length (approximately 2,000 meters). The sectors 00, 03, and 05 were previously disregarded for the study, either because the area’s relief favors a strong aircraft noise attenuation or because the aircraft fly over the homes at higher altitudes. Within the sectors 01, 02, and 04, the fifteen measuring sites were chosen in a similar way to that described by Carvalho et al.^5^ (2014), who adopted the identification of “sensitive points” (activities of public or collective nature, compatible with the residential use, such as education and health).

The measurements were carried out between May and October 2009. Each site was monitored for at least seven consecutive days without interruption. The data obtained on that occasion still represent the current situation, since neither the urban organization nor the operational airport characteristics showed modifications that may interfere with the noise sources behavior in the surrounding areas. The airport continues to operate since 2007 with the same characteristics that limit its operation in parameters inferior to both the demand and the installed capacity. Vehicle traffic, in turn, will only suffer modifications that might reduce the respective noise emission when completed all the infrastructure works that are in progress at the moment, particularly the Monorail Line 17-Gold – Morumbi-Jabaquara.

Five noise monitoring stations with meters and analyzers Larson Davis 824A plus accessories were used, which operate without permanent assistance or presence, resulting in three measuring campaigns, of five sites each. The meters were programmed to record all the measurement parameters derived from the dB(A) allowed by the equipment^f^, as well as to store all instantaneous noise samples, taken at intervals of one second, to allow the elaboration of other parameters calculations, when necessary.

The equipment also has been programmed to detect the characteristics of duration and loudness of aircraft overflights (takeoffs and landings), in each site, and to register the noise produced separately from the total noise measurement, which are very peculiar when compared with other mobile sources. Each record of this kind, called “event” by the memory of the equipment, had its instantaneous taken at every second, allowing the calculation of LEq and, mainly, the Sound Exposure Level (SEL)^7^ of each overflight, detected by crossing the data with the Control Tower reports, provided by the Airport administrator (Infraero). As Congonhas is an airport that operates a little diversified fleet, so that more elaborate segregation processes are not required^2^ ^,^ ^6^.

The SEL values of aircraft events were accumulated in periods of one hour and, after measurements, subtracted logarithmically from the SEL values of total noise, to obtain the background noise of each period. We considered the aircraft events recorded between 6:00 and 12:00, on Sunday, the most relevant results, because this period tends to be the one with less traffic noise influence in the Congonhas Airport vicinities. This leads to a better isolation of aircraft events, resulting in a more accurate calculation of the respective SEL and that, in turn, can be extrapolated to other periods in the remaining days of the week, when the background noise rises, making more difficult the noise events identification and segregation. As the operation of small aircraft (with capacity up to 30 passengers) is limited to only four operations per hour (landings plus takeoffs), for them, the same SEL average value was considered among the ones registered by the noisiest aircraft models. Approximately 80.0% of the air traffic is held with 320 series Airbus aircraft and the 737 NG Boeing family, very similar in size and noise emission^g^.

The sampling period of seven consecutive days was chosen because it captures all relevant events in a city’s routine. After this period, the environmental noise levels tend to repeat. Thus, it is possible to achieve good preliminary diagnostics without extending the measurements. Data analysis of noise from each monitored site considered the regulations of the Brazilian National Standards Organization (ABNT)^e^, which diagnoses the situation without using opinion surveys (more subjective).

Finally, the respective Meteorological Aerodrome Reports (METAR), elaborated by the air traffic control, were analyzed to exclude from the samplings the periods that showed bad weather conditions for the noise measuring by NBR 10151^d^ (wind above 9 km/h or rain) or short-term reversals in the takeoff and landing path directions.

## RESULTS

The Table summarizes the results for the fifteen sites monitored. Each site, of the 168 hours effectively carried out, obtained an average of 70 valid measurement hours. Two factors reduced the samples: the daily closing period of the airport runway and the periods of bad weather conditions (rain or wind that reversed the runway operation direction). Among the valid measurements it was observed that the background noise showed values above the ones recommended by national regulations in seven of the fifteen sites (points 11, 21, 25, 28, 29, 48, and 49). These sites are near major avenues and are exposed to significant noise, generated by cars and motorcycles seeking alternative paths to main roads, usually congested.

**Table:** Summary of monitoring results – Congonhas Airport.

Measuring site	No. of hours of the sample	LEq of total noise (dB/hour)	Min LEq – max LEq (dB–hour)	Average of overflights per hour	LEq of background noise (dB/hour)	Average of nominal differences (dB)	Remarks
Sector 01							
11 – Anne Frank Municipal Library	56	61.3	53.8–65.5	14	55.8	5.4	Exclusively residential area. Background noise 1 dB(A) above limit.
13 – São Germano Clinic	56	59.9	53.8–64.1	14	57.3	2.6	Area of mixed use. Total noise in the limit allowed.
12 – EMEF Mary Aux. D´Alquimin Bastos	71	65.3	59.5–67.6	15	58.1	7.2	Site right below the runway axis. Area of mixed use. Moderate to intense traffic.
Sector 02							
23 – EE Napoleão de Carvalho Freire	59	60.6	54.8–64.1	14	56.5	4.1	Exclusively residential area. Background noise 1.5 dB(A) above the limit.
27 – Franciscano Nossa Senhora Aparecida School	58	54.7	42.5–63.7	14	54.7	ZERO	Measured during school winter recess. Area of mixed use. Aircraft noise is blocked by nearby buildings.
25 – Pinheiros Orthopedic Clinic	45	61.4	56.5–64.4	18	56.5	4.8	Area of mixed use. Cobblestones pavement. Moderate to intense traffic.
21 – Brandão Educational Center	118	62.6	53.4–72.9	16	61.8	0.8	Area of mixed use. Relief favorable to reduction of aircraft noise. Background noise 1.8 dB(A) above the limit.
26 – Augusto Laranja School	112	65.5	59.9–70.8	16	62.4	3.1	Exclusively residential area. Site right below the runway axis. Background noise 2.4 dB(A) above the limit.
28 – Ibirapuera University	118	63.8	58.6–69.7	16	62.5	1.3	Area of mixed use with commercial predominance. Intense bus traffic. Background noise 2.5 dB(A) above the limit.
29 – EMEF Chiquinha Rodrigues	113	60.7	52.3–69.1	16	57.0	3.7	Area of mixed use. Cobblestones pavement. Moderate to intense traffic.

**Measuring site**	**No. of hours of the sample**	**Arithmetic mean of the total noise (dB/hour)**	**Min avg – Max avg (dB–hour)**	**Average of overflights per hour**	**Arithmetic mean of the background noise (dB/hour)**	**Average of nominal differences**	**Observations**

Sector 04							
48 – EMEF Armando de Arruda Pereira	64	62.9	58.8–70.8	18	58.7	4.2	Residential area. Relief unfavorable to noise attenuation. Site very sensitive between 19:00 and 21:00, when the heading is inverted. Background noise 3 dB(A) above the limit.
41 – Faculdade Colégio Montessori	70	65.7	53.8–71.1	17	64.8	0.9	Area of mixed use. Bus route. Moderate traffic. Measured during school winter holidays.
44 – Nossa Senhora de Lourdes Hospital	51	61.3	56.7–68.1	17	59.2	2.1	Area of mixed use. Site right below the runway axis. Moderate and disorganized access traffic to the hospital, even on weekends.
45 – Botanical Institute	70	53.7	41.2–63.1	16	ND	0	Aircraft taking off in high altitude. No vehicular traffic. Imperceptible/inaudible aircraft noise.
49 – Nossa Senhora das Graças School	21	67.1	61.6–79.5	17	62.9	4.2	Influence of highway’s noise. Small sampling because of the maintenance activity next to the equipment. Background noise 7 dB(A) above the limit.

EMEF: City School of Basic Education; EE: State High School; Min avg: average of the instantaneous minimum levels; Max avg: average of the instantaneous maximum levels; ND: not detected

In the eight remaining sites, four showed differences between aircraft noise and background noise lower than 3 dB(A) (points 13, 27, 41, and 45), caused by the existence of obstacles to aircraft noise propagation, by presenting favorable relief to attenuation or because the aircraft fly over the site at high altitudes. A distinct aircraft contribution to the annoyance was found only in the last four sites, due to their proximity to the airfield or the low altitude of overflights (points 12, 23, 26, and 44).

## DISCUSSION

**Figure 2:**
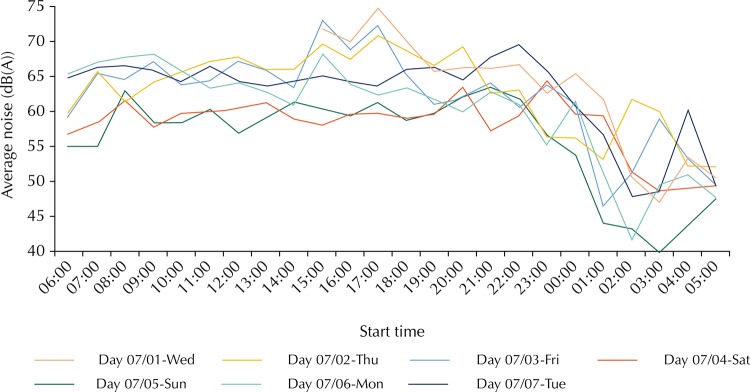
Behavior of one-hour LEq for total environmental noise (aircraft + background) in site #23.

Even in the sites where there was major contribution of aircraft noise to the total noise, it was observed that, between 9:00 and 21:00 pm, the one-hour LEq for total noise showed values above 65 dB(A), with the one-hour LEq for background noise above 60 dB (A). This happens due to the vehicle traffic noise (which increases with the number of vehicles in the streets), especially motorcycles^h^. From 2001 to 2012, the number of cars and light utility vehicles licensed in São Paulo doubled, while the motorcycle amount was multiplied by four. Another relevant fact is that the motorcycle models up to 175 cc (the most sold ones) can emit 3 dB(A) more noise than cars^i^. Figure 2 shows the one-hour LEq of total noise in one of the four sites with the greatest contribution to aircraft noise (site #23). It was observed that, even on Sundays, the total average noise was above the regulation limits (50 dB(A) between 7:00 and 21:59 and 45 dB(A) between 22:00 and 6:59).

A strong decrease in the total environmental noise can be observed after 21:00 in almost all sites measured. This phenomenon can be explained not only by the airport activity reduction (between 21:00 and 23:00), but also by the decrease of vehicle traffic on alternative routes, even though the main avenues present high volumes. At night, drivers tend to stay in the main corridors for security reasons (better lighting, policing, etc.), which shows the potential auxiliary role of airport noise monitoring to both determine noise pollution in relation to the day period^4^, and search the peculiar characteristic changes of the environmental noise within the airport closing limits^17^.

Thus, it was possible to observe interesting facts that make feasible to design additional mitigating measures, even at airports that already suffer high operational restrictions, such as Congonhas. In site #23, separated to be discussed in this article, there is a public school in an exclusively residential area presenting low traffic noise. The accordance with the noise regulation limits was only complied between 1:00 and 6:00. As the airport activity ceases daily at 23:00, it is possible to infer that the background noise is high not only in this period, but also during the whole day.

**Figure 3:**
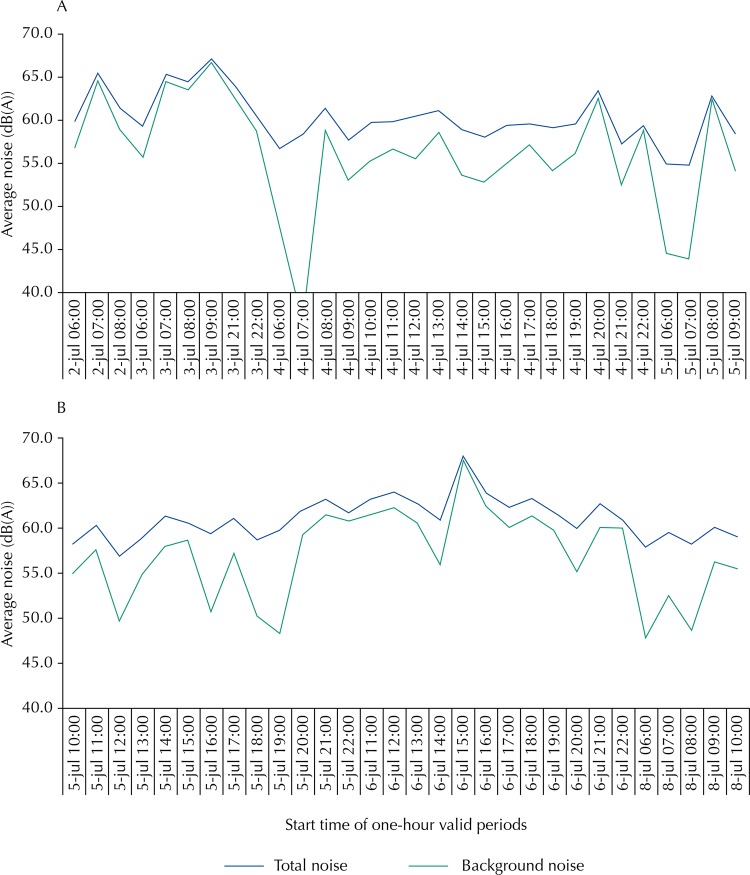
Nominal differences between environmental noise and background noise (one-hour LEq) for the site #23: 3A – part 1, 3B – part 2 (discontinuous sampling).

On July 5th, 2009, Sunday, between 6:00 and 11:00, the equipment programming to detect aircraft events registered 31 occurrences with instantaneous levels above the environmental noise level for more than 22 consecutive seconds, which were assigned to aircraft in landing procedure. Considering all the valid sampling, an average of 14 overflights were recorded per hour, by calculating an average SEL dB(A) of 81.6 for each overflight and 59 hours of valid measurement. By subtracting the aircraft SEL in each valid monitoring hour from the respective total environmental SEL, and then dividing the result by 3,600 seconds, one finds the background noise LEq (average) shown in Figure 3.

The average background noise exceeds by 6 dB(A) the regulation limit in many of the one-hour valid periods (Figure 3, green line), and the differences compared with the average total noise (Figure 3, blue line) are big (larger than 3 dB (A)), which means a strong influence of the airport operation on noise pollution, at this particular site. The highest differences between the total noise and the background noise were found in the airport closing limits, in the last two hours of operation at night (between 21:00 and 23:00) and mainly in the early morning (between 6:00 and 8:00), when the background noise is still low in intensity and the airport starts its operation with “full power.”

Since 2007, Congonhas Airport’s range is limited to attend destinations up to 1,000 km, which favors turboprop aircraft operations, quieter and more efficient in fuel consumption. Although they take less passengers and payload than the B737 NG and A320 aircraft, it is possible to think about a fleet replacement in the last and first hours of the airport operation period, if there is a lower demand for transportation^18^. Consequently, the differences between aircraft noise and background noise would be reduced, thereby reducing the sound impact. In a higher or lower degree, this approximation between background and aircraft noise was present in the other fourteen sites monitored for this study.

Therefore, the way the background noise is in the Congonhas Airport surroundings, it is possible that this urban equipment operates between 7:00 and 21:00 in its current configuration, without generating higher sound impacts. This daily “window” remains independently of seasonal variations as occurs, for example, in South Korea^12^ and Poland^14^. Still, the diagnostic parameters recommended by NBR 13368^e^ were efficient enough to dismiss the use of survey questionnaires applied to the population, which constitute more subjective sources of information.

However, the total environmental noise is a disturbing situation, because the national regulations of noise pollution are disrespected in almost all hours of the day, no matter the day of the week, being the airport open or closed. That means, even if the Congonhas Airport was “shut down” permanently, the problem of noise pollution would continue in its vicinities.

Much of the background noise registered is related to the increased circulation of motorcycles in the last ten years, which have been used by the inhabitants to replace deficient public transportation. This fact may spoil public health in several other aspects, because the regulation allows motorcycles to emit more noise and air contaminants than other vehicles. In addition, motorcycles are increasing emergency care costs, once the number of traffic accidents involving motorcycles also increased. It is, therefore, a vicious circle that contributes to degrade the population’s health.

## Footnotes

a International Civil Aviation Organization. International Standards and Recommended Practices. Annex 16: environmental protection. Vol. 1: Aircraft noise. Quebec: International Civil Organization; 2001.

b International Civil Aviation Organization. DOC 9829: Balanced approach guidance. Montreal: International Civil Aviation Organization; 2007.

c Agência Nacional de Aviação Civil. RBAC 161: planos de zoneamento de ruído. Brasília (DF): Agência Nacional de Aviação Civil; 2012. Preconiza valores de LDN para as curvas interna e externa de, respectivamente, 75 e 65. (cited 2015 jan 15). Available from: http://www.anac.gov.br/assuntos/legislacao/legislacao-1/rbha-e-rbac/rbac/rbac-161-emd-01/@@display-file/arquivo_norma/RBAC161EMD01.pdf

d Associação Brasileira de Normas Técnicas. NBR-10151: Avaliação do ruído em áreas habitadas visando o conforto da comunidade. Rio de Janeiro (RJ): Associação Brasileira de Normas Técnicas; 2000.

e Associação Brasileira de Normas Técnicas. NBR-13368: Ruído aeronáutico - Monitoramento. Rio de Janeiro (RJ): Associação Brasileira de Normas Técnicas; 1995.

f Sound level (LEq), sound exposure level (SEL), percentile levels (Ln), maximum (Lmax) and minimum sound level (Lmin), among others.

g Both aircraft are classified by Annex 16 Vol 1 ICAO as Chapter 4 (jet aircraft with maximum takeoff weight higher than 5,700 kg and whose prototype was certified after 2001).

h Rodrigues JM. Evolução da frota de automóveis e motos no Brasil, 2001-2012: relatório 2013. Rio de Janeiro (RJ): Instituto Nacional de Ciência e Tecnologia, Observatório das Metrópoles; 2013 (cited 2015 Jan 15). Available from: http://www.observatoriodasmetropoles.net/download/auto_motos2013.pdf

i Conselho Nacional do Meio Ambiente. Resolução 272: Limites de emissão de ruído para veículos novos. Brasília (DF): Conselho Nacional do Meio Ambiente; 2000.
